# Chromosome instability and aneuploidy as context-dependent activators or inhibitors of antitumor immunity

**DOI:** 10.3389/fimmu.2022.895961

**Published:** 2022-07-15

**Authors:** Xiaohong Kuang, Jian Li

**Affiliations:** ^1^ Department of Hematology, The Third Hospital of Mianyang, Sichuan Mental Health Center, Mianyang, China; ^2^ Department of General Surgery, The Third Hospital of Mianyang, Sichuan Mental Health Center, Mianyang, China

**Keywords:** chromosome instability, aneuploidy, antitumor immunity, immune evasion, tumor evolution

## Abstract

Chromosome instability (CIN) and its major consequence, aneuploidy, are hallmarks of human cancers. In addition to imposing fitness costs on tumor cells through several cell-intrinsic mechanisms, CIN/aneuploidy also provokes an antitumor immune response. However, as the major contributor to genomic instability, intratumor heterogeneity generated by CIN/aneuploidy helps tumor cells to evolve methods to overcome the antitumor role of the immune system or even convert the immune system to be tumor-promoting. Although the interplay between CIN/aneuploidy and the immune system is complex and context-dependent, understanding this interplay is essential for the success of immunotherapy in tumors exhibiting CIN/aneuploidy, regardless of whether the efficacy of immunotherapy is increased by combination with strategies to promote CIN/aneuploidy or by designing immunotherapies to target CIN/aneuploidy directly.

## Introduction

The propensity of cells to produce daughter cells with numerically and/or structurally altered chromosomes compared with their parent cells is known as chromosome instability (CIN). Various cellular alterations can lead to CIN, including incorrect kinetochore-microtubule attachments, dysfunction of the spindle assembly checkpoint (SAC), supernumerical centrosomes and multipolar spindle formation, premature loss of cohesion, and stress responses such as DNA replication, oxidative, proteotoxic and mechanical stresses (1, 2). The major outcome of CIN is the emergence of cells with aneuploidy, referred to as cells with a total number of chromosomes that is not a multiple of the normal haploid complement. In addition, CIN is linked to both structural and numerical chromosomal aberrations, including polyploidy, chromosomal translocation, genome chaos, as well as different types of nonclonal chromosome aberrations (NCCAs) (3). CIN and aneuploidy are not identical: CIN describes a high propensity for chromosome gain and loss, while aneuploidy represents a state with an imbalanced karyotype (4). Although the definition of aneuploidy have not reached a consensus in the literature, more recently, to investigate the importance of aneuploidy in tumorigenesis and for practical reasons, Ben-David U and Amon A strongly encourage the field to adopt the definition of aneuploidy as copy number alterations (CNAs) that affect either entire chromosome arms (excluding the short arms of acrocentric chromosomes) or whole chromosomes. Such a uniform definition would increase consistency and reproducibility across cancer studies (5). In this review, we combined them as CIN/aneuploidy unless otherwise specified. However, unlike gene mutations, by which the cellular changes imparted are easily identifiable, it is difficult to predict the phenotypic functions of CIN/aneuploidy, as hundreds to thousands of genes are affected simultaneously. One class of consequences of CIN/aneuploidy is determined by the specific karyotype and specific genes located on them. Another class of consequences consists of the general effects of unbalanced genomes independent of karyotype, including growth defects, genomic instability, endoplasmic reticulum (ER) stress, and metabolic deficiencies (1, 2). The phenotypic changes are also shaped by the microenvironment in which aneuploid cells reside (1, 2).

The notorious phenotypic change in humans attributed to CIN/aneuploidy is malignant transformation. Aneuploidy has long been viewed as a hallmark of tumors, with 90% of solid tumors displaying some degree of aneuploidy, and the majority of human cancers, irrespective of their origins, exhibit CIN (6). CIN/aneuploidy is associated with tumor progression and is a biomarker of poor prognosis in several human tumor types (6, 7). Despite the pervasiveness of CIN/aneuploidy in tumors, their exact roles in cancer are complex and still inconclusive. In the view of somatic evolution, tumors are shaped by the complex interaction of clonal expansion, genetic diversification and clonal selection (8). Large-scale genomic analysis of tumor samples indicates that CIN provides a punctuated burst of heterogeneous aneuploid subclones, followed by clonal expansion of the fitted aneuploid clones but elimination of the unfitted karyotypes (9). However, except for in certain healthy tissues, such as neurons and primary hepatocytes, many studies have shown that aneuploidy rarely occurs in normal tissues even when the fidelity of chromosome segregation is lost (10–12). Euploid stem cells have been shown to outcompete aneuploid stem cells in mosaic embryos (13). These findings suggest the possibility that cells with CIN/aneuploidy may lose viability *in vivo*. Meanwhile, findings in cancer research showed that aneuploidy acts both oncogenically and as a tumor suppressor, and low levels of CIN promote tumor initiation and progression, but higher levels are protective and suggest that highly aneuploid, chromosomally unstable tumors may exhibit enhanced sensitivity to aneugenic or DNA-damaging drugs (14). For years, studies performed to explain such unfitness have mainly focused on cell-intrinsic mechanisms. As mentioned above, CIN/aneuploidy imparts several cellular burdens, leading to growth arrest or death of tumor cells. Therefore, aneuploid cells senesce prematurely and grow poorly as tumor xenografts (15, 16). However, numerous studies published in recent years have demonstrated that these aberrant cells may also be eliminated by microenvironmental factors, especially by antitumor immune responses.

Here, we review the evidence obtained from various contexts showing the paradoxical interplay between CIN/aneuploidy and tumors. We further discuss the mechanistic basis underlying the activation of the antitumor immune response by CIN/aneuploidy and how tumor cells evolve to overcome such activation. Finally, we provide insights into possible explanations for that disparity and the clinical potential of leveraging CIN/aneuploidy to increase the efficacy of immunotherapy for tumors.

## Induction of antitumor immune response activation

**Figure 1:**
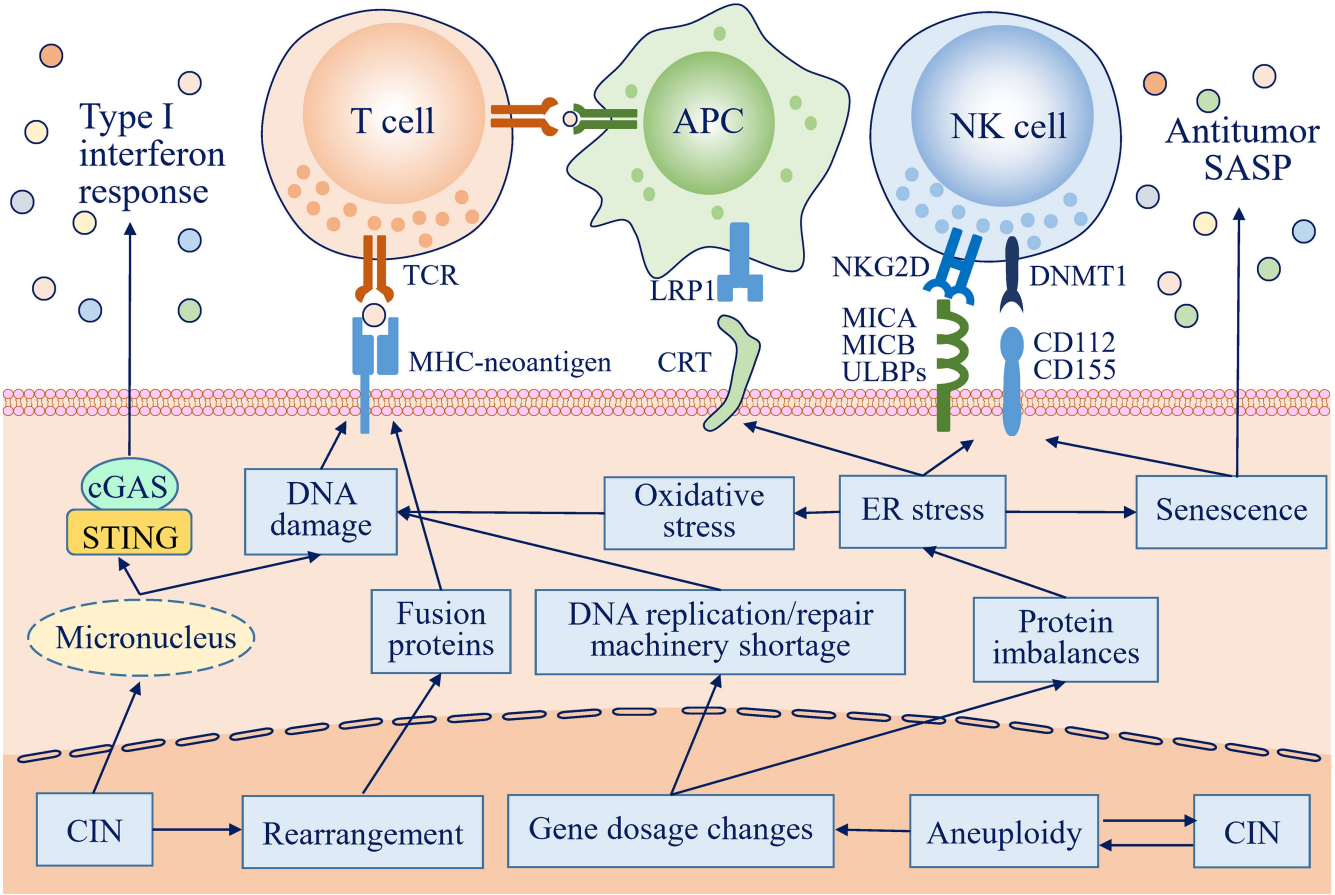
Antitumor immune responses can be activated by chromosome instability (CIN) and aneuploidy in many ways. In cells with CIN, lagging chromosomes form micronuclei, which are prone to rupture, and DNA is then exposed to the cytosol to activate the cGAS-STING pathway, further inducing an antitumor type I interferon response. Gene dosage changes resulting from CIN/aneuploidy lead to protein imbalances, followed by endoplasmic reticulum (ER) stress. ER stress increases the expression of activating natural killer (NK) cell ligands and redistributes endogenous calreticulin (CRT) to the surface of the plasma membrane, which are recognized by and activate NK cells and antigen-presenting cells (APCs), respectively, to eliminate transformed cells by innate and adaptive immune responses. ER stress also promotes cells with CIN/aneuploidy to acquire a senescence phenotype and to secrete a broad range of mediators to create an antitumor immunoenvironment. In addition, DNA damage resulting from a replication/repair machinery shortage, exposure to the cytosol, and oxidation, along with complicated DNA rearrangement, can increase the immunogenicity of CIN/aneuploid cells by creating many potential neoantigens.

In animal models, xenografts with induced ploidy changes tended to form tumors in immunocompromised mice but failed to grow or grew more slowly in immunocompetent mice, and cancer cells with hyperploidy induced by microtubular poisons protected immunocompetent mice from rechallenge with the same live cancer cells (17, 18). Histological examination of tumors revealed that the mean nuclear diameter was decreased in immunocompetent mice compared with tumors arising from immunocompromised mice, and the DNA contents were also reduced compared with those of cancer cells cultured *in vitro* (17). This evidence suggests that antitumor immune responses can be activated by CIN/aneuploidy, and cancer cells will face the selection pressure imposed by the immune system (**Figure 1**).

### Activation of the cGAS-STING pathway

Under normal circumstances, DNA is strictly confined to the nucleus by the nuclear envelope to prevent it from becoming a damage-associated molecular pattern (DAMP); otherwise, it is sensed by cyclic GMP-AMP synthase (cGAS)/the stimulator of interferon genes (STING) pathway and activates the innate immune system in the cytosol and endosomal compartments (19). In cells with high levels of aneuploidy, the cGAS-STING pathway was found to be upregulated (20). As the cGAS-STING pathway is intimately associated with the immune response, the activation of the immune system in cells with CIN/aneuploidy may partly result from the activation of the cGAS-STING pathway (21). How does CIN/aneuploidy induce a cytosolic DNA cGAS-STING pathway response? cGAS was found to localize with micronuclei, and interferon-stimulated gene (ISG) expression was mainly observed in micronucleated cells by combining live-cell laser microdissection with single-cell transcriptomics, indicating that DNA within micronuclei is the major contributor (22, 23). A common feature in cells with CIN/aneuploidy is the increased number of lagging chromosomes during anaphase and micronuclei in the following G1 (20). Attributed to the defective nuclear lamina organization, irreversible nuclear envelope collapse was observed in up to 60% of micronuclei, leading to the exposure of micronuclear DNA to the cytosol (24). The direct association between chromosome mis-segregation and activation of the cGAS-STING pathway was also supported by chromosome-tracking experiments, which demonstrated that the mis-segregated chromosomes ultimately become fragmented cytoplasmic chromatin (25). The exposed DNA is recognized by and activates cGAS and then produces the cyclic dinucleotide cGAMP, which in turn activates the adaptor STING to induce a type I interferon response and activate NF-κB, mainly by inducing the phosphorylation and subsequent activation of interferon regulatory factor 3 (IRF3) (26).

### Enhanced killing by natural killer cells

Natural killer Group 2 member D (NKG2D) ligands, such as major histocompatibility complex-class I chain-related proteins A and B (MICA/B) and UL16-binding proteins (ULBPs), the main natural killer (NK) cell activating ligands, were shown to be highly expressed in cells with complex karyotypes or hyperploidy; the same is true for DNAX accessory molecule-1 (DNAM1) ligands CD112 and CD155 but not for the NK-cell inhibitory human leukocyte antigen (HLA) class I molecules (17, 20). These cell-surface ligands mediate the recognition and activation of NK cells, triggering clearance of cells with CNI/aneuploidy. Therefore, when these cells were cocultured with NK cells, the rates of recognition and destruction were much higher than those in euploid cells, while cytotoxicity was abolished by NKG2D and DNAM-1 blocking antibodies (17, 20, 27). The increased expression of NK cell-activating ligands may be contributed to the ER stress and DNA damage responses caused by CNI/aneuploidy, as inhibition of protein kinase RNA-activated-like ER kinase (PERK) and ataxia-telangiectasia mutated (ATM) were shown to inhibit the induction of MICA on the surface of hyperploid cells (17, 28, 29). In addition, the senescence-associated secretory phenotype (SASP) gene expression pattern of aneuploid-induced senescent cells was shown to elicit NK cell recognition and elimination of cancer cells *in vivo* (6). Moreover, coculture with hyperploid cancer cells has been shown to significantly increase the proliferation of NK cells, mainly through the indirect induction of IL-2 secretion by other lymphocytes (17). These effects were also supported by clinical findings. Serum IL-2 and NK cell activities were significantly increased in patients with advanced breast cancer following treatment with taxanes, which inhibits microtubule polymerization to induce CIN/aneuploidy (30).

### Increased DAMP release

When hyperploidy was induced in primary or malignant murine and human cell lines or organoids by cytokinesis or microtubule inhibitors, endogenous calreticulin (CRT) was found to redistribute to the surface of the plasma membrane (17). Additionally, in immunocompetent mice, the rechallenge protection role of cancer cells with hyperploidy was strongly reduced by depletion of CRT but was restored by regain recombinant CRT (17). The depletion of CTR also mitigated the differences in the growth of cancer cells in immunodeficient versus immunocompetent mice (17). The role of CRT in activating the antitumor immune response depends on signaling through the pattern recognition receptor (PRR) low-density lipoprotein receptor-related protein 1 (LRP1), which promotes phagocytosis by phagocytes and induces the secretion of cytokines that enhance neoantigen presentation (31). The reasons associated with CRT redistribution have not been completely elucidated. However, the introduction of only one additional copy number of chromosome 7 did not induce such CRT redistribution unless further aneuploidy was caused by cytochalasin D, indicating that only the levels of aneuploidy beyond a physiological level can stimulate CRT exposure (17). Therefore, CRT redistribution may be a result of the ER stress response because microtubule perturbation-induced exposure of CRT at the cell surface was accompanied by PERK-associated phosphorylation of eukaryotic initiation factor 2α (eIF2α), upregulation of X-box binding protein 1 (XBP1), and perinuclear translocation of activating transcription factor 6 (ATF6) (17). In contrast, depletion of factors involved in the ER stress response reduced the transportation of CRT to the plasma membrane in aneuploid cells (17). ER stress was also found immunohistochemically in tetraploid Tp53−/− colon organoids (18). Other DAMPs released by ER stress, high mobility group box 1 (HMGB1) and ATP, induce proinflammatory cytokine production by dendritic cells (DCs) and macrophages through the toll-like receptor 4 (TLR4) receptor for advanced glycation end-products (RAGE) and purinergic receptor P2X7 or act as a “find me” signal; however, these effects have not been validated in cancer cells with CIN/aneuploidy (32–34).

### Induction of senescence and inflammatory mediator secretion

Following treatment by monopolar spindle 1 (MPS1) inhibitor in diploid cells, although 80% of cells with less than 5% of their genomes affected by genomic imbalances retained the ability to divide continuously, the induced highly aneuploid cells in which genomic imbalance affected more than 20% of their genomes tended to be senescent, manifesting higher numbers of γ-H2AX foci, elevated levels of the senescence markers p53, p21, and p16, and increased senescence-associated β-galactosidase activity (20). These highly abnormal karyotypes exhibit a SASP-like gene expression signature (20). Although other aneuploidy-related genomic alterations also likely contribute to senescence, mouse embryonic fibroblasts (MEFs) harboring specific trisomies that did not experience significant DNA damage also showed an SASP gene expression profile, indicating that aneuploidy per se can trigger a senescence phenotype (20). The SAPS consists of a broad range of secreted factors, which form a proinflammatory microenvironment and activate an antitumor immune response.

Even without senescence, cells with aneuploidy also showed increased expression of genes that mediate inflammation and immune response, even in primary cells harboring very low levels of aneuploidy (20, 35). The elevated gene set categories in highly aneuploid cells represented immune response gene expression signatures, including interferon-α/β signaling, graft versus host disease, antigen processing and presentation, and autoimmune/thyroid disease (20). In addition to the elevated expression of inflammatory genes, the secretion of cytokines and chemokines, including interleukin-6 (IL-6), IL-8, and C-C motif ligand 2 (CCL2), was also found to be increased (20). In fibroblasts, lymphoblastoid cells, circulating monocytes and T cells obtained from Down syndrome patients, the interferon pathway was consistently activated, which may have resulted from the increased gene dosage of interferon receptor genes located on chromosome 21 (36). In cells with high levels of aneuploidy, the phosphorylation of STAT3 and SAPK/JNK was higher, suggesting that the secreted inflammatory mediators can trigger a feedforward loop (20).

### Shape the tumor antigenic landscape

Genomic alterations, especially point mutations and rearrangements, affect oncogenes and tumor suppressor genes and are the main causes leading to malignant transformation. These genomic alterations also produce neoantigens, which can be recognized by immune cells, resulting in the elimination of transformed cells. Although the tumorigenic role of CIN was mainly hypothesized to be the increased or decreased expression of oncogenes and/or tumor suppressor genes by chromosome gain or loss, and CIN/aneuploidy and DNA damage are closely interrelated and can directly cause or be caused by one another, positive correlations between the mutation burden and aneuploidy levels are significant for the number of mutations only in passenger genes and not in cancer driver genes. This result indicates that the association is unidirectional and that DNA damage indeed occurs during CIN to produce an immunogenic change in protein sequence (37). γ-H2AX, a marker of DNA damage, was increased in micronuclei and found to be transiently and modestly increased 3-6 hours after chromosome mis-segregation, both indicating that the creation of micronuclei during CIN may induce DNA damage (20, 23). In another study, the majority of cells with induced aneuploidy could proliferate continuously without cell cycle arrest by p53 activation. However, approximately 10-15% of cells showed p53 activation and G1 arrest, which was proposed to be the result of DNA damage accrued during chromosome mis-segregation (20). Therefore, in aneuploid cells, p53 can be activated by ATM, a DNA damage checkpoint kinase (38). Several mechanisms may contribute to the DNA damage arising from CIN/aneuploidy. First, lagging chromosomes can be trapped in the cytokinetic furrow, which may cause DNA damage (39, 40). Second, a shortage of DNA replication and repair machinery in micronuclei or resulting from gene copy number changes in primary nuclei, along with premature anaphase onset induced by CIN, leads to incompletely replicated or decatenated DNA, resulting in segregation of damaged DNA into progeny cells (20, 41, 42). Third, the envelope of micronuclei is unstable and tends to rupture, which exposes chromosomes to cytoplasmic nucleases, resulting in DNA damage (24, 43). Fourth, DNA damage can be caused by aneuploidy-related stresses, such as oxidative stress, which produces reactive oxygen species (ROS), leading to DNA damage (38). Finally, chromosomes of cells with CIN/aneuploidy are prone to break near repetitive sequences, leading to chromosome rearrangements or even highly complex chromothripsis (44, 45). Genomic rearrangement has the potential to generate multiple frameshift mutations, which are stronger triggers of antitumor T cell reactivity (46).

Despite these findings supporting the notion that CIN and aneuploidy are important contributors to genomic instability and DNA damage, the mutation burden related to CIN/aneuploidy is inconclusive; additionally, studies on the effects of CIN/aneuploidy on mutation are limited, especially those on the production of neoantigens with the ability to stimulate an immune response. In a pancancer study based on genomic data, a cancer genome hyperbola was found; one tumor had either a large number of aneuploidies or a large number of somatic mutations, but never both (47). In contrast, another pancancer study based on the same genomic data but with different inclusion criteria demonstrated a positive correlation between the mutation burden and aneuploidy level in the majority of cancer types (37). The controversies may be multifactorial and need to be validated in various contexts, especially the effects of CIN/aneuploidy on the production of neoantigens, which have important clinical significance.

### Increased tumor antigen presentation and activation of the adaptive immune response

Although the evidence is limited, CIN/aneuploidy increases the redistribution of CRT on the plasma membrane of cancer cells, which may act as an “eat-me” signal for antigen-presenting cells (APCs), leading to the presentation of tumor antigens to and activation of T cells (17). Following treatment with docetaxel, which can induce aneuploidy by stabilizing microtubules, the surface expression of CRT on cancer cells was elevated, causing immunogenic modulation but without immunogenic cell death, suggesting an increased sensitivity to antigen-specific cytotoxic T lymphocyte (CTL) killing (48). Furthermore, after the injection of aneuploid cancer cells, mice that did not develop tumors exhibited restricted growth from another inoculation with parental but not unrelated cancer cells, indicating that aneuploidy can activate protective adaptive immune responses (17). Indeed, when CD4+ or CD8+ T lymphocytes were depleted or when interferon-γ or type I interferon receptor 1 was inactivated in these mice, the ability to form tumors of hyperploid cells was increased (17). Compared with their parental counterparts, hyperploid cells were also shown to be more efficient at priming T cells against tumor antigens (17). In addition, the cGAS-STING pathway activated by CIN/aneuploidy may be involved in the antitumor adaptive immune response, which has been found to provoke a CD8+ T cell response against cancer (49, 50). STING was also found to be essential for radiotherapy in combination with immune checkpoint inhibitors (ICIs) to elicit an abscopal effect, which requires T cell responses, indicating that CIN/aneuploidy may activate an adaptive immune response through the cGAS-STING pathway (23, 51).

## Overcome antitumor immune activation

Although it supports an immunogenic role of CIN/aneuploidy, this evidence was mainly obtained from experiments directly manipulating the ploidy of tumor cells. In contrast, in clinical scenarios, CIN/aneuploidy is a hallmark of many tumors. Metastasis persistence and recurrence exhibited no evidence of immnoediting and tended to be hyperploidy (52). The gene expression signatures associated with adaptive immunity, cytotoxic activities mediated by CD8+ T and NK cells, an ongoing immune response and a cytokine-rich microenvironment were significantly downregulated in tumors with a highly aneuploidy phenotype, and the responsiveness to ICIs was decreased in melanomas associated with aneuploidy (37, 53). Additionally, the degree of aneuploidy was correlated with markers of immune evasion (37, 53–55). These findings suggest that at some point, cancer cells acquire the ability to tolerate chromosome segregation errors and utilize the continuous evolution caused by CIN to overcome antitumor immune activation.

**Figure 2:**
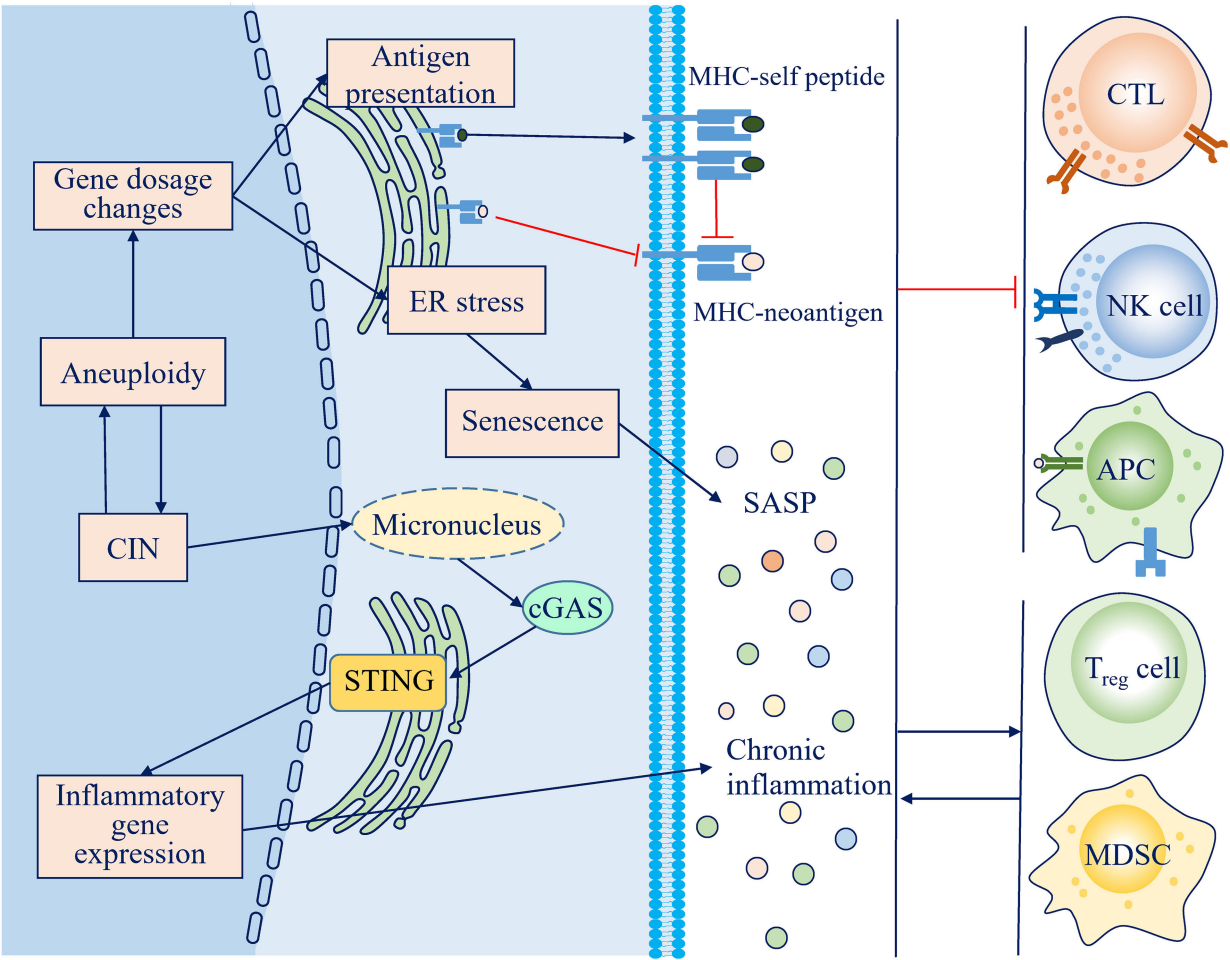
Mechanisms through which chromosome instability (CIN) and aneuploidy escape the antitumor immune response. Persistent activation of the cGAS-STING pathway and the senescence-associated secretory phenotype (SASP) lead to a chronic inflammatory microenvironment, which impairs the antitumor immune response and facilitates an immunosuppressive microenvironment. Loss of neoantigens and antigen presentation machinery during CIN, along with competitive inhibition by self-peptides attributed to protein imbalance, decreases the immunogenicity of CIN/aneuploid cells.

Immune evasion is a long-accepted hallmark of tumors, contributing to tumor formation and progression (56). However, the mechanisms by which CIN induces the transition from immune detection to immune evasion are not well known and remain to be elucidated. Cancer has long been considered as a somatic evolutionary problem. Internal and external environment imposes stress on every cell dynamically, new phenotypes are needs to emergency for viability. However, cellular adaptation requires genetic and epigenetic changes and, as a trade-off, it leads to genome alterations. When the new genome becomes dominant, it can break the system constraints of the higher level (57). For cancer cells, immunosurveillance is a forceful stress, which may induce the emergency of new genome in cancer cells with the ability to break the constraints of immune system. The tumor immune evasion state is established through the selection of tumor subclones resistant to an immune attack mediated by cooperation between innate and adaptive immune responses (56). CIN/aneuploidy and related cellular stresses may be involved in every pathway that participates in this selection (**Figure 2**). Tumor cells utilize the versatility of CIN in a context-dependent manner to minimize the lethal consequence of antitumor immune response activation while inducing an immune-privileged tumor microenvironment (TME) to sustain tumor progression.

### Loss of neoantigens and their presentation

One immune evasion mechanism mediated by CIN is to deplete the expression of neoantigens and antigen presentation machinery. In non-small-cell lung cancer (NSCLC), copy number loss affects more chromosome loci with more neoantigenic nonsynonymous mutations than with less neoantigenic nonsynonymous mutations, leading to subclonal loss of previously clonal neoantigens and low immune infiltration (58). Compared with their near-diploid parental cells, tumors can grow well in immunocompetent mice and were found to decrease the expression of genes involved in antigen presentation (59). CIN-induced loss of heterozygosity (LOH) is the main way by which tumor cells achieve a decrease in neoantigen presentation. In NSCLC, 40% of tumor cells were found to have copy-number LOH in the HLA locus, which was associated with increased T-cell infiltration but a high subclonal neoantigen burden, suggesting immunoselection for aneuploid cancer cells that fine-tune their major histocompatibility complex I (MHC I) dosage (60). In serous ovarian cancer, similar LOH was mainly found in tumor regions with CD8+ T cell repletion, suggesting dynamic spatial selection (61). Somatic LOH at the HLA locus has also been observed in human melanoma and is associated with a worse response to ICIs and poor prognosis (62). In addition to the loss of neoantigen and antigen presentation machinery, the decrease in effective antigen presentation may involve the relative concentration of neoantigen. The average neoantigen concentration in hyperploid tumors is lower than that in near-diploid tumors, as the increase in unstable proteins resulting from the imbalanced stoichiometry of many protein complexes generates more self-peptides, which may compete for limited MHC proteins (37).

### Suppression of the cGAS-STING pathway or type I interferon signaling

As the main activator of the antitumor immune response, the cGAS-STING pathway has been extensively studied in tumors with CIN/aneuploidy; therefore, successfully established tumors must evolve ways to avoid its activation or hijack its activation for tumor progression. Consistent with this notion, the cGAS-STING pathway is frequently inactivated in tumors, and tumor cells are often unable to induce type I interferon signaling by transfected cGAMP or dsDNA (63, 64). The protein levels of cGAS and STING were lower in colorectal cancers and melanoma with advanced stages, while the reduction in STING mMRA and protein levels correlated with increased stages in gastric cancer (63, 65, 66). However, genes encoding cGAS and STING are rarely found to be inactivation-mutated or copy-number-altered, indicating that epigenetic silencing may play an important role (7, 67). Nonetheless, studies have revealed that some tumors indeed retain or even increase the expression of cGAS and STING, indicating that under specific conditions, activation of the cGAS-STING pathway and its corresponding expression of genes involved in inflammation and the immune response are still preserved but function as tumor promoters (68, 69). In other circumstances, activation of the cGAS-STING pathway by CIN/aneuploidy is not synonymous with the induction of type I interferon signaling. In tumor cells, ongoing stress resulting from CIN and endogenous DNA damage activate the p38 pathway, leading to inhibition of interferon signaling downstream of STING (70). The loss of the interferon gene cluster on chromosome 9p during CIN may be another mechanism underlying the lack of a type I interferon response to cytosolic DNA, which has been observed in many cancer types (7, 71, 72). Beyond inhibition of the type I interferon response, activation of the cGAS-STING pathway alternatively induces other inflammatory pathways, including noncanonical NF-κB (25). Noncanonical NF-κB was observed to promote the progression of tumors directly or inhibit the expression of type I interferons indirectly (73). Therefore, tumor cells with low CIN formed fewer metastases than their CIN-high counterparts, while depletion of STING decreased the tumor burden and metastatic dissemination of CIN-high cells (25).

### Induction of protumor inflammatory mediators

In contrast to the induction of antitumor cytokines mentioned above, in immune evasion stages, CIN/aneuploidy can also promote the secretion of inflammatory mediators to support cancer progression. In a mouse model of colon cancer, CIN caused by haplosufficient Shugoshin-1 leads to an increased expression of protumorigenic, proinflammatory factors such as cyclooxygenase-2 (COX-2) and IL-6 (74). Another mouse model of CIN-induced tumorigenesis is associated with chronic inflammation mediated by NF-κB (75). Genomic data of human tumors showed that aneuploidy positively correlates with the activation of inflammatory pathways and overall leukocyte infiltration but is dominated by immune-suppressive macrophages and the activation of tumor growth factor-β (TGF-β) (76). Under certain circumstances, senescent cells secrete a broad range of inflammatory mediators to promote the progression of neighboring tumor cells. After transformation by an oncogenic activating RAS, human fibroblasts showed mitotic spindle and chromosome aberrations and featured senescence effector p21 and p16 expression, suggesting associations between cell survival and CIN-induced senescence (77). SASP-related inflammatory mediators recruit immune cells to the TME, which in turn propagate CIN through genotoxic stress or the induction of mesenchymal-epithelial transition (EMT), maintaining a feed-forward cycle beneficial for tumor evolution (78, 79).

## Reconcile disparity: context is everything

### Evolution stages determine the effects of CIN/aneuploidy

**Figure 3:**
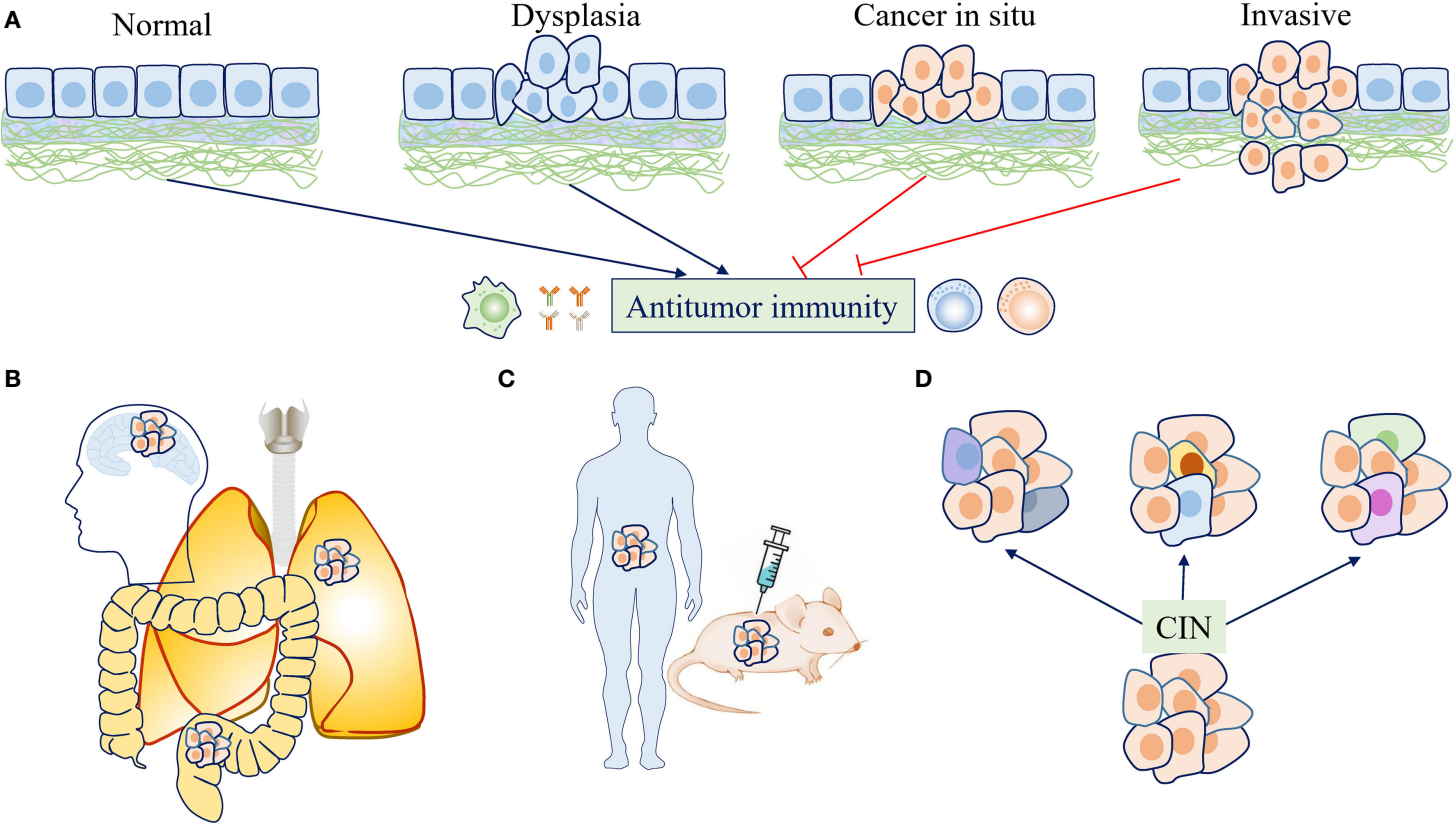
The effects of chromosome instability (CIN) and aneuploidy on antitumor immunity are context dependent. **(A)** At the early stage of tumor evolution, aneuploid cells were restrained by immunosurveillance. If the immune system has not eliminated these transformed cells completely, persistent CIN may continually produce “new” karyotypes, allowing the selection of cancer cells with the ability to escape immunosurveillance. **(B)** Tumors with different origins or locations may face different immune backgrounds and exhibit different immune infiltration, which plays a role in determining the effects of CIN/aneuploidy on antitumor immunity. **(C)** Various study designs and experimental conditions may lead to different findings regarding the effects of CIN/aneuploidy on antitumor immunity. **(D)** CIN drives the development and maintenance of intratumor heterogeneity (ITH) as a stochastic generator for diverse karyotypes, which may also have different effects on immune responses due to karyotype-specific consequences or different dosage alterations of gene expression.

Multiple genomic alterations lead to malignant transformation, while these alterations also initially activate cell-autonomous and extrinsic immune-mediated antitumor mechanisms. Therefore, the transformed cells must evolve to overcome these barriers for unrestrained growth. In a recently published study, aneuploid colon cancer cells were injected subcutaneously into mice, and the initial growth was significantly worse in immunocompetent mice than in immunodeficient mice (59). However, following evolution for several generations, the selected cells exhibited superior growth even in immunocompetent mice (59). The results indicate that CIN thus seems to be able to promote both immune detection and immune evasion, depending on the tumorigenic stage (5). At the early stage of tumor evolution, aneuploid cells are restrained by immunosurveillance; therefore, under the pressure of immunoselection, only the fittest clones can be chosen to form a tumor successfully (80). A novel karyotype caused by CIN may be the most effective way to increase the fitness of transformed cells because rapid genome-wide changes are more effective than other types of genomic instability to supply cells with new phenotypes under different stress conditions (81). Therefore, if the immune system has not eliminated these transformed cells completely, persistent CIN may serve as a stochastic generator for genomic instability, continually producing “new” karyotypes and increasing intratumor heterogeneity (ITH), allowing the selection of cancer cells with the ability to escape immunosurveillance. Patients with tumors are generally diagnosed at advanced stages, and cancer-based clinical analyses always identify the abovementioned results, as clinically diagnosed cancers have already subverted immune surveillance (**Figure 3A**). However, what time points within the tumor evolution trajectory the CIN/aneuploidy change their roles in cancer immunesurveillance is not clear at present, as accumulating evidence found that aneuploidy can be found in preneoplastic lesions and even in clones in phenotypically normal tissues, which were associated significantly increased cancer risk (82, 83). There have no studies investigate the involvement of cancer immunesurveillance in these conditions, which warrants further studies.

### Tumor location dictates the immune response

The normal immune microenvironment varies across organs or different parts and tissues within the same organ (84, 85). Therefore, tumors with different origins or locations may face different immune backgrounds and display different immune infiltration (86–88). Such diversity may also play a role in determining the effects of CIN/aneuploidy on the immune system (**Figure 3B**). For example, the expression of genes in signatures characteristic of the antitumor immune response was significantly decreased in epithelial tumor types and melanomas but not observed in brain cancers such as glioblastoma multiforme (GBM) and lower-grade glioma (LGG) (37). Furthermore, cancer cells that formed brain metastases expressed cGAMP as a result of cytosolic DNA-mediated cGAS-STING activation (89). Although this observation was explained by the passage of cGAMP from cancer cells to neighboring astrocytes through gap junctions, it is possible that in an immunoprivileged microenvironment, cGAS-STING activation cannot elicit an antitumor response but instead supports the survival of tumor cells (89). As many tumor types were shown to be “immune cold”, the inflammatory response elicited by CIN/aneuploidy through cGAS-STING pathway activation may actually play a tumor-promoting role. Therefore, when immunotherapy was combined to provoke an effective antitumor response, STING agonists have been shown to reduce tumor growth (90, 91). In addition, tumor origin may also influence the effects of CIN/aneuploidy on the mutation burden. Although the majority of cancer types display a positive correlation between aneuploidy and the number of mutations, a statistically significant negative correlation was found in colorectal carcinoma (CRC) and uterine corpus endometrial carcinoma (UCEC), which was mainly dependent on the presence of hypermutated samples (37). Although these differences may be contributed to the fact that CIN and other genomic instability are two distinct tumorigenesis pathways, the direct associations between them cannot be excluded. Another intriguing finding is that CIN/aneuploidy are prevalent in specific normal organs, such as the liver and brain. However, in these physiological conditions, CIN/aneuploidy are not associated with tumorigenesis but are beneficial for normal function development or a mechanisms to mediate stress-induced adaptation (10–12). To elucidate how these organs to tolerate the aneuploidy-targeted immunosurveillance will help shedding light on how aneuploid cancer cells evolve to overcome the antitumor immune activation.

### Heterogeneity in study design and experimental conditions

A note of caution should be kept in mind when interpreting and extrapolating the results of the studies mentioned in this review; specifically, there are fundamental differences among cultured tumor cells, tumors in animals developed by injection or carcinogen induction, and naturally occurring spontaneous tumors in humans (**Figure 3C**). For example, the protein structure and reactivity to DNA of human cGAS differ significantly from those of its mouse counterpart (92). Tumor-establishing methods such as injection and chemical carcinogens, even tissue contaminants, can induce an immune response (93). Mouse tumor models in experiments are always established in rapid ways, while tumorigenesis in humans is a slow process that lasts for several years. The same pathway may elicit different immune responses under these different conditions. For example, the mechanisms underlying different types of inflammatory downstream activation of cGAS-STING under different conditions are still poorly understood. However, the duration of inflammation may be an important factor. At the establishment stage of cancer or in experiments observing short-term outcomes, CIN/aneuploidy mainly induces acute cGAS-STING signaling, which activates the antitumor type I interferon response and SASP. However, when they escape elimination by the host, established tumors induce a persistent inflammatory microenvironment, and chronic engagement of the cGAS-STING pathway and its corresponding SASP represent a driver of tumor progression (25, 94).

### Intratumor heterogeneity

ITH has been studied extensively in recent years due to advances in single-cell sequencing technologies, and was found to be important for cancer progression (95). CIN drives the development and maintenance of ITH as a stochastic generator for diverse karyotypes, which may also have different effects on immune responses due to karyotype-specific consequences or different dosage alterations of gene expression (**Figure 3D** **)**. For example, the immune signature is more accurately predicted by arm/chromosome somatic copy number alteration (SCNA) events than by focal SCNA events (37). Although focal SCNA is not considered aneuploidy, the differences in immune signature prediction may reflect the consequences of different dosage alterations of gene expression or specific affected genes. Such an association may be the result of immunoselection, but the possibility of shaping by CIN/aneuploidy cannot be excluded.

## Perspectives on leveraging CIN/aneuploidy for cancer immunotherapy

**Figure 4:**
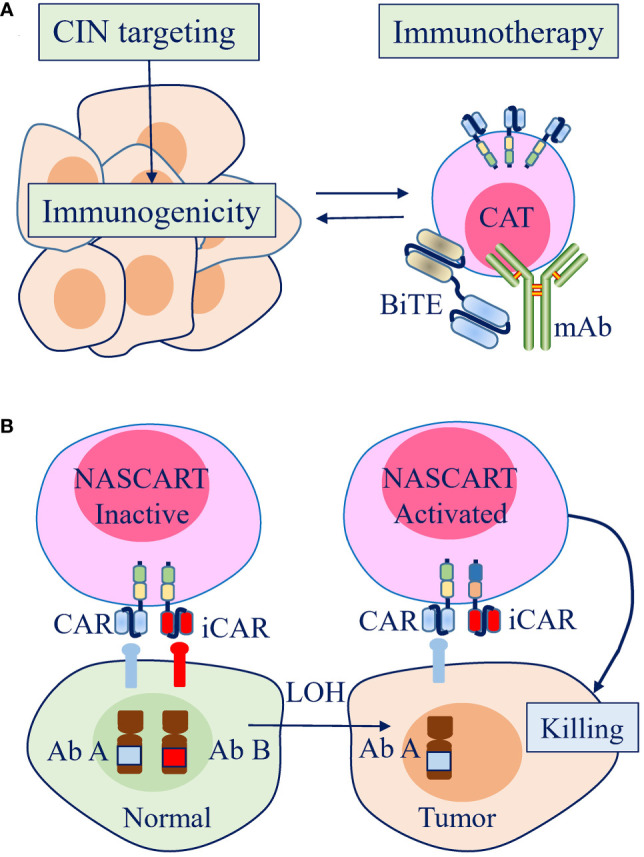
Therapeutic strategies to leverage chromosome instability (CIN) and aneuploidy to enhance the efficacy of antitumor immunotherapy in the clinic. **(A)** Strategies promoting CIN can increase the immunogenicity of tumor cells, which can be combined to enhance the efficacy of antitumor immunotherapy. **(B)** A pairwise chimeric antigen receptor (CAR) platform, Neoplasm-targeting Allele-Sensing CAR (NASCAR), has the potential to target loss of heterozygosity (LOH), which is mostly contributed by CIN/aneuploidy. The activating and inhibitory CARs were designed to target polymorphic forms of the same molecule or to target different molecules, but both with the inhibitory molecules were lost by LOH in tumor cells.

Due to their complex roles in tumors, designing therapies to target CIN/aneuploidy is difficult, especially targeting them to manipulate the immune response. Under the current understanding of the role of CIN/aneuploidy in tumors, two approaches are feasible. The first approach is to increase the antitumor immune response by manipulating CIN/aneuploidy through combination therapies. Another approach is to design immunotherapies that directly target CIN/aneuploidy or its consequences (**Figure 4**).

### Increasing the antitumor immune response by targeting CIN/aneuploidy

Excessive CIN/aneuploidy leads to fitness costs, and tumor cells must restrict CIN within a limited rate that maintains their viability. Disruption of this balance to promote CIN was the main basis on which to design therapies targeting CIN and partly contributed to the effects of currently available antitumor therapies. CIN/aneuploidy activates antitumor immune responses through the mechanisms mentioned above, indicating that local or systemic antitumor immunity also underlies the effects of these therapies. For example, cGAS-STING signaling and antitumor immunity are important determinants of the tumor response to taxol, PARP inhibitors, and radiation (23, 96, 97). Therefore, the combination of CIN/aneuploidy-inducing drugs with ICIs provides interesting therapeutic potential. For example, in a murine colon cancer model, cooperative effects were found between CFI-402257, a selective Mps-1 inhibitor, and an anti-PD1 antibody (98). Unfortunately, CIN has long been appreciated to facilitate tumor progression and treatment resistance by inducing ITH at the level of gene dosage. Therefore, when a cancer is diagnosed in humans, CIN/aneuploidy has always evolved mechanisms to overcome the antitumor role of the immune system, even converting the immune system to be tumor-promoting (99). These findings have important implications for patient selection and therapy design. First, immunotherapy should be best combined at an early stage of tumor development, when host antitumor immunity is still preserved and overcoming mechanisms have not been established. Second, as mentioned above, the effects of the immune response also depend on the duration and degree of CIN/aneuploidy, leading us to question the role of the long-term combination of cytotoxic therapies in immunotherapy. Third, as a source of ITH to support resistance, CIN/aneuploidy should be better induced before immunotherapy to improve response rates; however, continued CIN should be mitigated during treatment, which may be crucial to avoid acquired resistance. Fourth, according to the model of two-phased cancer evolution, which comprises a punctuated phase and a gradual stepwise phase, CIN plays an important role within the punctuated phase and is responsible for phase switching. Therefore, monitoring treatment using CIN to reduce induced genome chaos-mediated drug resistance is the key for the success of immunotherapy targeting CIN/aneuploidy (57, 100).

### Immunotherapy directly targeting CIN/aneuploidy

CIN/aneuploidy is defined as extensive in denotation but lacks specificity, which contradicts the concept of targeted therapy design. However, targeting LOH, which is contributed to most by CIN/aneuploidy, emerges as a possible strategy. Recently, a pairwise chimeric antigen receptor (CAR) platform, Neoplasm-targeting Allele-Sensing CAR (NASCAR), was established (101). The activating and inhibitory CARs were designed to target polymorphic forms of the same molecule or to target different molecules, but both with the inhibitory molecules were lost by LOH in tumor cells. Thus, NASCAR T cells will be activated in tumors in which only activating molecules exist, while they will remain inactivated in normal tissues due to the presence of inhibitory molecules (101). Although this approach remains in its infancy and has limitations, given that the sparsity of tumor-specific antigens presents a substantial obstacle for the wide application of powerful immunotherapy, targeting LOH may be a promising approach because it is one of the frequent somatic alterations in human tumors.

## Conclusions

The tumor immune microenvironment is a major determinant of the outcome of cancer. In this review, we have focused on how the antitumor immune response is regulated by CIN/aneuploidy, a frequent hallmark of human tumors. As discussed in this review, controversy and context dependence are the only two conclusions that can be made unambiguously. Despite recent advances in our understanding of the role CIN/aneuploidy plays in antitumor immunity, the associated clinical application remains in its infancy, and there are still many unknowns requiring further research. Addressing CIN/aneuploidy in antitumor immunity is essential for the success of personalized therapy, a problem that is only just beginning to be understood. As the effects of CIN/aneuploidy on antitumor immunity are context dependent, careful patient selection and therapy design will undoubtedly be central to the success of antitumor immunity by targeting CIN/aneuploidy. Furthermore, determining when and how tumor cells evolve mechanisms to overcome the antitumor role of the immune system or convert the immune system to be tumor-promoting will be key to our ability to design strategies to target them for a therapeutic benefit.

## Author contributions

XK wrote the first version of the manuscript. JL provided critical feedback and additions. JL prepared the figures. All authors contributed to the final version of the manuscript and approved the submitted version.

## Funding

This study was supported by Scientific Research Projects of Health Commission of Mianyang City (202012).

## Conflict of interest

The authors declare that the research was conducted in the absence of any commercial or financial relationships that could be construed as a potential conflict of interest.

## Publisher’s note

All claims expressed in this article are solely those of the authors and do not necessarily represent those of their affiliated organizations, or those of the publisher, the editors and the reviewers. Any product that may be evaluated in this article, or claim that may be made by its manufacturer, is not guaranteed or endorsed by the publisher.
